# Outcomes and prognostic factors of repeat pulmonary metastasectomy

**DOI:** 10.1093/icvts/ivae028

**Published:** 2024-02-29

**Authors:** Ryu Kanzaki, Hirokazu Watari, Akiisa Omura, Sachi Kawagishi, Ryo Tanaka, Tomohiro Maniwa, Jiro Okami

**Affiliations:** Department of General Thoracic Surgery, Osaka International Cancer Institute, Osaka, Japan; Department of General Thoracic Surgery, Osaka International Cancer Institute, Osaka, Japan; Department of General Thoracic Surgery, Osaka International Cancer Institute, Osaka, Japan; Department of General Thoracic Surgery, Osaka International Cancer Institute, Osaka, Japan; Department of General Thoracic Surgery, Osaka International Cancer Institute, Osaka, Japan; Department of General Thoracic Surgery, Osaka International Cancer Institute, Osaka, Japan; Department of General Thoracic Surgery, Osaka International Cancer Institute, Osaka, Japan

**Keywords:** Pulmonary metastasis, Surgery, Repeat resection

## Abstract

**OBJECTIVES:**

Information on prognostic factors after repeat pulmonary metastasectomy (PM) is limited, and outcomes after a third PM are not well documented.

**METHODS:**

A single-institute retrospective study was conducted. Between 2000 and 2020, 68 patients underwent repeat PM for pulmonary metastases from various cancers. Outcomes and prognostic factors for the second PM and outcomes after the third PM were analysed.

**RESULTS:**

This study included 39 men and 29 women. The mean age at second PM was 53.2 years old. The primary tumours were soft tissue sarcoma in 24 patients, colorectal cancer in 19 and osteosarcoma in 10. The interval between the first PM procedure and detection of pulmonary metastasis after the first PM (months) was ≤12 in 37 patients and >12 in 31 patients. At the second PM, 20 patients underwent lobectomy or bilobectomy, and 48 underwent sublobar resection. Complete resection was achieved in 60 patients, and 52 patients experienced recurrence after the second PM. The 5-year relapse-free survival and overall survival rates after the second PM were 27% and 48%, respectively. Multivariable analysis revealed that the interval between the first PM and the subsequent detection of pulmonary metastasis (≤12 months) was a poor prognostic factor for both relapse-free survival and overall survival after the second PM. Seventeen patients underwent a third PM, 3 of whom achieved a 3-year disease-free survival.

**CONCLUSIONS:**

Patients with a period of >12 months between the first PM and the subsequent detection of pulmonary metastases showed favourable outcomes and are thus considered good candidates for second PM. A third PM may be beneficial for selected patients.

**Figure:**
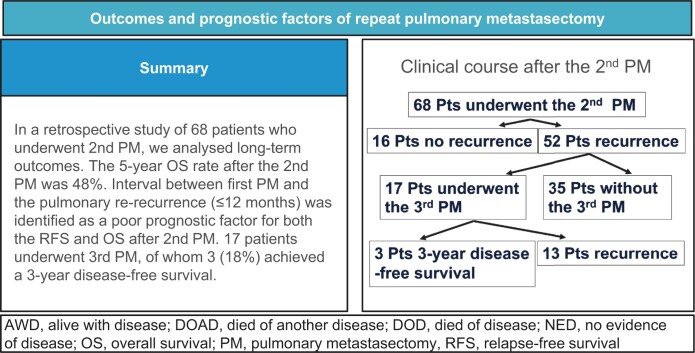

## INTRODUCTION

Despite the lack of evidence from randomized trials, pulmonary metastasectomy (PM) is regarded as a viable treatment option for patients with pulmonary metastasis from various types of cancers [1, 2]. Since first mentioned by Thomford *et al.* [3], several modifications have been made with regard to the principal criteria for the indication of PM, and Kondo *et al.* [4] summarized these criteria in the current era. Since 2000, multidetector-row computed tomography (CT) has become widely used. Because thin-section CT provides a more accurate count of the number of pulmonary metastases than conventional CT [5], the indication for PM has been judged more precisely since the introduction of multidetector-row CT.

A certain proportion of patients who undergo PM experience pulmonary recurrence after the first PM procedure and meet the criteria for repeat PM. The PM procedure is performed a second time in these patients, following which favourable outcomes have been reported in various types of cancers [1, 2, 6–10]. However, while there have been many reports on prognostic factors associated with the survival after first PM, information on prognostic factors after repeat PM is limited [2].

A relatively small percentage of patients who underwent a second PM and showed re-recurrence met the criteria for a third PM procedure. In the report of The International Registry of Lung Metastases, which included 5206 cases of PM, 15% of patients underwent repeat PM, and only 5% underwent PM ≥3 times [11]. Krüger *et al.* reported that 35 of 621 patients (6%) who underwent first PM subsequently underwent third PM [12]. Recently, Mills *et al.* [13] reported the short-term outcomes of third PM, concluding that the third PM can be performed safely and feasibly in select patients. However, there has been little information on the mid- to long-term outcomes after third PM. In this clinical context, information on the prognostic factors for repeat PM and mid- to long-term outcomes after the third PM is needed.

Therefore, we analysed the outcomes and prognostic factors for second PM, as well as the outcomes and clinical course after the third PM, based on 2 decades of experience in our institution.

## PATIENTS AND METHODS

Between 2000 and 2020, a total of 599 patients underwent PM for pulmonary metastases originating from malignancies other than primary lung cancer at the Osaka International Cancer Institute. After excluding 50 patients with insufficient data or a follow-up period of <3 months, 549 patients were analysed in this retrospective study. Among these patients, 68 patients underwent repeat PM and this cohort was subjected to a further analysis. It is noteworthy that the planned staged bilateral pulmonary resection was regarded a single PM procedure in the present study and was not included as a repeat PM. Patients who underwent surgical biopsy were also excluded from this study. Preoperative diagnoses of pulmonary nodules were made on the basis of chest CT findings. In all patients, the primary tumours were pathologically diagnosed prior to pulmonary resection, and the primary tumour received treatment that included surgery or heavy ion radiotherapy. In all cases, lung specimens were histologically evaluated and pulmonary metastases were diagnosed by pathologists. Clinical information was collected from the medical records at our hospital.

In our institution, the mode of treatment is determined by a multidisciplinary cancer board, which includes thoracic surgeons, medical oncologists, radiation oncologists and primary tumour specialists such as colorectal surgeons, orthopaedic surgeons and head and neck surgeons. Patients generally underwent PM after meeting the following criteria [4]: (i) complete resection of the pulmonary metastasis (or metastases) was considered achievable; (ii) the metastatic lesions were limited to the lungs, or extrapulmonary distant metastases were already controlled if present; (iii) the patient’s primary tumour was already controlled or controllable; (iv) lymph node metastasis from the pulmonary lesion was determined to be absent on preoperative evaluation; and (v) the general condition of the patient was good, and the patient’s respiratory function was sufficient to tolerate pulmonary resection. Repeat PM was performed if the patient met the criteria for the first PM. The type of resection and surgical approach were selected according to the size and location of recurrent pulmonary metastases. In our institution, intraoperative lavage cytology was routinely performed to analyse the surgical margins, as previously described [14].

The indications for perioperative chemotherapy and the timing of chemotherapy were determined by the surgeons or physicians in charge after considering the extent of the disease and the general condition of the patient.

Follow-up was generally based on chest X-ray or chest CT, physical examination and blood chemistry evaluations, which were performed every 3–6 months after the first PM.

In the survival analysis of patients with pulmonary metastases after the first PM to compare the survival of patients who underwent a second PM to that of patients who did not undergo a second PM, overall survival (OS) was defined as the time interval between the detection of pulmonary recurrence after the first PM and death from any cause. The significance of differences between 2 groups was calculated using the generalized Wilcoxon test. For further survival analysis of patients who underwent a second PM, OS was defined as the time interval between the second PM and death by any cause, and relapse-free survival (RFS) was defined as the time interval between the second PM and the first recurrence of primary tumour cancer or death due to any cause. The follow-up period was defined as the interval between the date of pulmonary resection and the date of death or latest follow-up. The data cut-off date was September 10, 2022. The median follow-up times after the first and second PM in the present study were 46 (range: 3–273) and 36 (range: 3–220) months, respectively.

Statistical analyses were performed using JMP Pro 14.2.0 software program (SAS Institute, Berkley, CA, USA). The data are expressed as mean ± standard deviation or median values. RFS and OS after pulmonary resection were analysed using the Kaplan–Meier method. The Cox constant proportional hazards model was used to assess the effects of the covariates on OS and RFS. Statistical significance was set at *P* < 0.05. Factors with *P* values <0.05 in the univariate analysis were used for the subsequent multivariable analysis.

The study protocol was approved by the Institutional Review Board of the Ethics Committee of Osaka International Cancer Institute (control number 22113).

## RESULTS

The primary tumours of 549 patients who underwent first PM are shown in Supplementary Material, Table S1. Colorectal cancer (CRC) in 192 (35%), head and neck cancer in 74 (13%) and soft tissue sarcoma (STS) in 74 (13%) patients. Of these, 313 patients (57%) experienced recurrence. Among these, 68 patients underwent a second PM (with second PM group) and 68 patients experienced lung-limited recurrence and did not undergo a second PM (without second PM group). The remaining 177 patients experienced recurrence other than lung recurrence or recurrence at both the lung and other sites and did not undergo a second PM. The treatment modalities in 68 patients who experienced lung-limited recurrence and did not undergo a second PM were as follows: chemotherapy (*n* = 42), other systemic therapy (*n* = 3), radiotherapy (*n* = 6), radiofrequency ablation (*n* = 5), chemoradiotherapy (*n* = 1), best supportive care (*n* = 8) and unknown (*n* = 3). The OS of patients with second PM and those without second PM group after the detection of pulmonary recurrence after the first PM are shown in Supplementary Material, Fig. S1. The OS of patients with second PM was significantly better than that of patients without second PM (5-year OS: 53% vs 41%, *P* = 0.01).

A further analysis of 68 patients who underwent a second PM was conducted, and the characteristics of these patients are shown in Table 1. The mean age at the time of primary tumour treatment was 48.3 years. The primary tumours were STS in 24 (35%) patients, CRC in 19 (28%) and osteosarcoma in 10 (15%). Sixty-seven patients (98%) underwent treatment including surgery for the primary tumour. The interval between treatment for the primary tumour and the first PM was none (synchronous) in 5 (7%), 1–24 months in 33 (49%) and >24 months in 30 (44%) patients. The interval between the first PM procedure and the subsequent detection of pulmonary metastasis (months) was ≤12 months in 37 patients (54%) and >12 months in 31 patients (46%).

**Table 1:** Patient characteristics

Characteristics	Number of patients (*n* = 68)
Sex, *n* (%)	
Male	39 (57%)
Female	29 (43%)
Age at treatment for primary tumour (years)	
Mean ± SD	48.3 ± 20.0
Range	13–77
Primary tumour, *n* (%)	
Soft tissue sarcoma	24 (35%)
Colorectal cancer	19 (28%)
Osteosarcoma	10 (15%)
Renal cell carcinoma	4 (6%)
Oesophageal cancer	2 (3%)
Head and neck cancer	4 (6%)
Germ cell tumour	2 (3%)
Others	3 (4%)
Treatment mode for primary tumour, *n* (%)	
Treatment includes surgery	67 (98%)
Heavy ion radiotherapy	1 (2%)
Interval between treatment for primary tumour and first PM (months), *n* (%)	
0 (synchronous)	5 (7%)
1–24	33 (49%)
>24	30 (44%)
Interval between first PM and subsequent detection of pulmonary metastasis (months), *n* (%)	
≤12	37 (54%)
>12	31 (46%)
Metastasis aside from the lung before the time of second PM, *n* (%)	
Yes	24 (35%)
No	44 (65%)
Preoperative chemotherapy for second PM, *n* (%)	
Yes	28 (41%)
No	40 (59%)

PM: pulmonary metastasectomy; SD: standard deviation.

The surgical factors for the first and second PM are listed in Table 2. The rate of solitary metastasis was higher in the second PM (53% in the first PM vs 75% in the second PM). In the second PM, 46 patients (68%) underwent surgery on the ipsilateral side of the first PM, that is they underwent redo thoracotomy on the same side. The rate of anatomical resection (i.e. segmentectomy or lobectomy) was high in second PM (26% in first PM vs 44% in second PM). Furthermore, the rate of thoracotomy was high, the operation time was long, the blood loss volume was high and the rate of postoperative complications was high in the second PM. Complete resection was achieved in all 68 patients at the first PM and in 60 patients (88%) at the second PM. In terms of the surgical margin of lung parenchyma, intraoperative lavage cytology was performed and a negative margin was confirmed in 62 patients who underwent a second PM. The following postoperative complications occurred after second PM: prolonged air leak (*n* = 2), atrial fibrillation (*n* = 2), empyema (*n* = 2) and others (*n* = 4).

**Table 2:** Surgical factors at first and second pulmonary metastasectomy

Factors	First PM (*n* = 68)^a^	Second PM (*n* = 68)^b^
Age (years)		
Mean ± SD	51.1 ± 20.1	53.2 ± 20.3
Range	14–78	15–79
Location of pulmonary metastasis, *n* (%)		
Unilateral	54 (79%)	58 (85%)
Bilateral	14 (21%)	10 (15%)
Number of lesions, *n* (%)		
1	36 (53%)	51 (75%)
2	16 (24%)	8 (12%)
3	5 (7%)	4 (6%)
≥4	11 (16%)	5 (7%)
Size of pulmonary metastasis (cm)		
Mean ± SD	1.5 ± 0.9	2.3 ± 2.8
Range	0.4–4.1	0.3–20.0
Redo thoracotomy on the same side, *n* (%)	Not applicable	46 (68%)
Surgical approach, *n* (%)		
Thoracotomy	44 (65%)	54 (79%)
Video-assisted thoracic surgery	24 (35%)	14 (21%)
Type of resection, *n* (%)		
Lobectomy or bilobectomy	9 (13%)	20 (29%)
Segmentectomy	9 (13%)	10 (15%)
Partial resection	50 (74%)	38 (56%)
Operation time (min), mean ± SD	106 ± 58	144 ± 92
Blood loss (g), mean ± SD	75 ± 135	194 ± 331
Complete resection, *n* (%)	68 (100%)	60 (88%)
Postoperative complications, *n* (%)	6 (9%)	10 (14%)

a Data from the preceding pulmonary resection procedure were used for 6 patients who underwent two-stage pulmonary resection for bilateral metastases.

b In 6 patients who underwent two-stage pulmonary resection for bilateral metastases, data from the preceding pulmonary resection procedure were used.

PM: pulmonary metastasectomy; SD: standard deviation.

**Figure 1:**
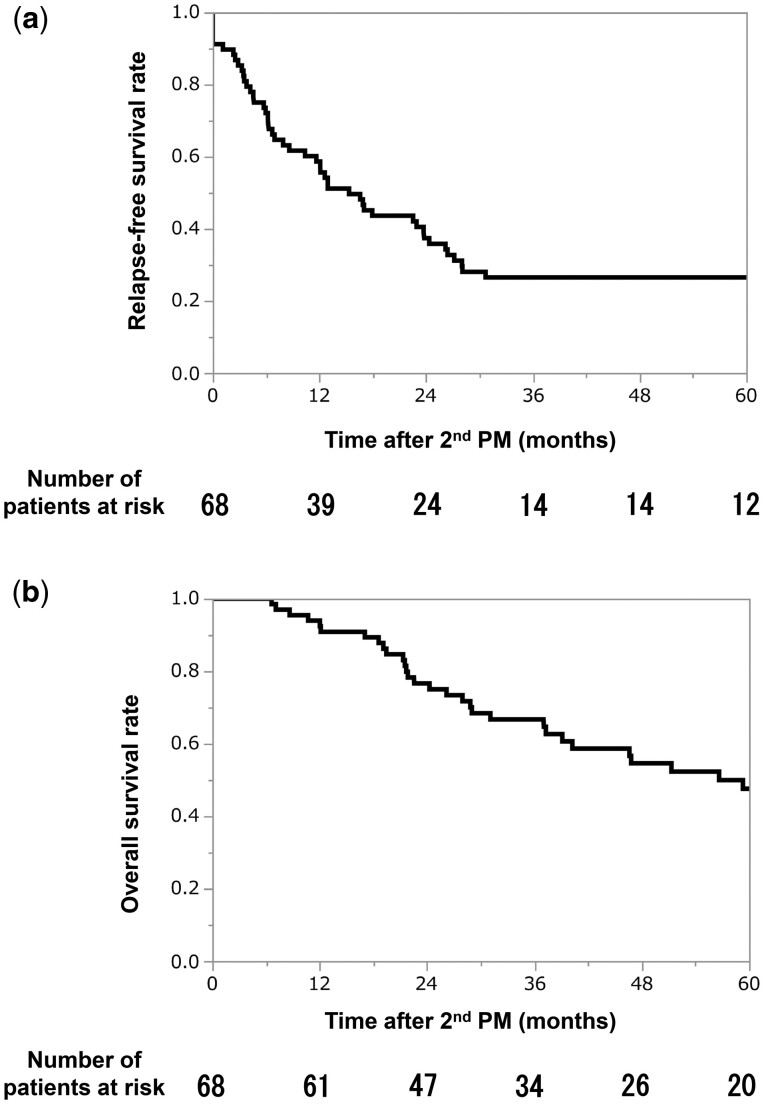
A survival analysis of 68 patients who underwent pulmonary metastasectomy a second time. PM: pulmonary metastasectomy. (**a**) Relapse-free survival after second pulmonary metastasectomy. (**b**) Overall survival after second pulmonary metastasectomy.

After the second PM, 52 patients (76%) experienced relapse. At the time of data cut-off, 29 patients were alive and 39 patients died. The 5-year RFS and OS rates after the second PM were 27% and 48%, respectively (Fig. 1a/b).

The factors influencing RFS after the second PM are shown in Table 3. Multivariable analysis showed that age (≤60 years), interval between the first PM and subsequent detection of pulmonary metastasis (≤12 months) and completeness of resection of the second PM (incomplete) were poor prognostic factors for RFS. The results of the analysis of the factors influencing OS after the second PM are shown in Table 4. Multivariable analysis showed that the interval between the first PM and the subsequent detection of pulmonary metastasis (≤12 months) was a poor prognostic factor for OS.

**Table 3:** Analyses of factors influencing the relapse-free survival after second pulmonary metastasectomy

Factor	Univariate analysis			Multivariable analysis		
	Risk ratio	95% CI	*P-*value	Risk ratio	95% CI	*P-*value
Age at second PM (>60/≤60 years)	0.45	0.26–0.78	0.0047	0.43	0.23–0.78	0.0061
Sex (male/female)	0.92	0.53–1.60	0.7628			
Histologic type (sarcoma/nonsarcoma)	2.28	1.32–3.93	0.0032	1.76	0.95–3.24	0.0709
History of distant metastasis besides lung before second PM (yes/no)	1.06	0.58–1.92	0.8527			
Interval between first PM and subsequent detection of pulmonary metastasis (≤12/>12 months)	2.72	1.55–4.78	0.0005	2.8	1.38–5.65	0.0042
Interval between detection of pulmonary metastasis after first PM and second PM (≤3/>3 months)	1.02	0.59–1.73	0.9548			
Interval between first and second PM (≤24/>24 months)	3.48	1.83–6.62	0.0001	1.38	0.53–3.64	0.5097
Chemotherapy before second PM (yes/no)	1.29	0.75–2.21	0.3584			
Number of metastases at second PM (multiple/solitary)	1.92	1.04–3.53	0.0366	1.87	0.79–4.42	0.1533
Location of pulmonary metastasis at second PM (bilateral/unilateral)	1.14	0.53–2.44	0.7412			
Size of tumour at second PM (>20/≤20 mm)	0.76	0.42–1.38	0.3728			
Sum of the number of metastases at first and second PM (≥3/2)	1.41	0.82–2.44	0.2121			
Sum of the number of metastases at first and second PM (≥4/≤3)	1.75	1.00–3.07	0.0498	1.47	0.64–3.40	0.3654
Type of resection for second PM (segmentectomy or lobectomy/wedge resection)	1.17	0.57–2.40	0.6657			
Operation time for second PM (≥120/<120 min)	0.81	0.47–1.41	0.4627			
Amount of bleeding at second PM (>50/≤50 g)	0.78	0.45–.1.36	0.3856			
Completeness of resection at second PM (incomplete/complete)	4.08	1.86–8.96	0.0005	6.81	2.75–46.85	<0.0001
Postoperative complication after second PM (yes/no)	0.78	0.33–1.82	0.5602			

CI: confidence interval; PM: pulmonary metastasectomy.

**Table 4:** Analyses of factors influencing the overall survival after second pulmonary metastasectomy

Factor	Univariate analysis			Multivariable analysis		
	Risk ratio	95% CI	*P-*value	Risk ratio	95% CI	*P-*value
Age at second PM (>60/≤60 years)	0.53	0.28–1.02	0.0571			
Sex (male/female)	0.72	0.38–1.37	0.3203			
Histologic type (sarcoma/nonsarcoma)	2.11	1.10–4.07	0.0256	1.69	0.86–3.33	0.1308
History of distant metastasis besides lung before second PM (yes/no)	0.97	0.47–2.00	0.931			
Interval between first PM and subsequent detection of pulmonary metastasis (≤12/>12 months)	3.78	1.83–7.81	0.0003	2.95	1.32–6.58	0.0082
Interval between detection of pulmonary metastasis after first PM and second PM (≤3/>3 months)	0.98	0.52–1.85	0.9583			
Interval between first and second PM (≤24/>24 months)	2.49	1.20–5.16	0.0139	1.5	0.66–3.39	0.3339
Chemotherapy before second PM (yes/no)	1.19	0.63–2.24	0.5956			
Number of metastases at second PM (multiple/solitary)	1.72	0.88–3.36	0.1112			
Location of pulmonary metastasis at second PM (bilateral/unilateral)	1.42	0.65–3.10	0.3845			
Size of tumour at second PM (>20/≤20 mm)	1.12	0.57–2.21	0.7329			
Sum of the number of metastases at first and second PM (≥3/2)	1.25	0.65–2.38	0.5081			
Sum of the number of metastases at first and second PM (≥4/≤3)	1.59	0.84–2.98	0.1532			
Type of resection for second PM (segmentectomy or lobectomy/wedge resection)	1.41	0.65–3.09	0.387			
Operation time for second PM (≥120/<120 min)	1.36	0.70–2.63	0.3589			
Amount of bleeding at second PM (>50/≤50 g)	1.3	0.68–2.49	0.4316			
Completeness of resection at second PM (incomplete/complete)	2.22	0.84–5.85	0.1064			
Postoperative complication after second PM (yes/no)	1.41	0.55–3.67	0.4757			

CI: confidence interval; PM: pulmonary metastasectomy.

**Figure 2:**
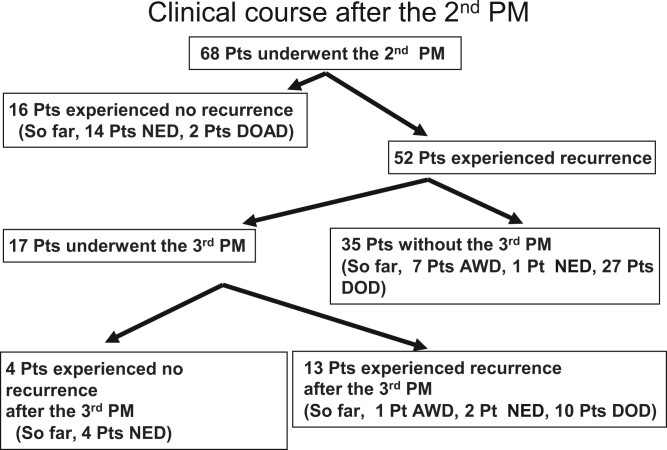
The clinical course after the first pulmonary metastasectomy procedure. AWD: alive with disease; DOAD: died of another disease; DOD: died of disease; NED: no evidence of disease; PM: pulmonary metastasectomy; Pts: patients.

The clinical course of the second PM is shown in Fig. 2. Among the 52 patients who experienced relapse after the second PM, the sites of relapse were pulmonary metastasis alone in 30, distant metastasis aside from the chest cavity in 6, pulmonary metastasis and pleural dissemination in 4, pleural dissemination alone in 3, mediastinal lymph node metastasis in 3, pulmonary metastasis and distant metastasis aside from the chest cavity in 3, local relapse of the primary tumour in 2 and pulmonary metastasis and surgical margin relapse of pulmonary metastasis in 1 patient.

**Figure 3:**
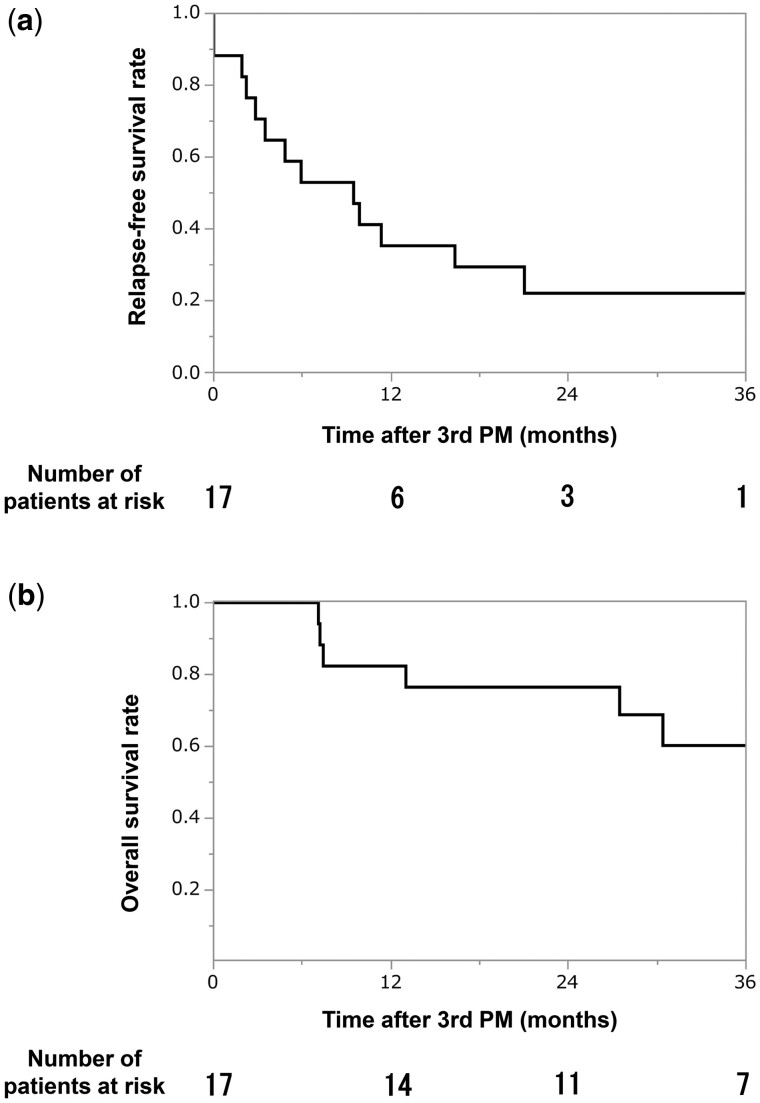
A survival analysis of 17 patients who underwent pulmonary metastasectomy a third time. PM: pulmonary metastasectomy. (**a**) Relapse-free survival after third pulmonary metastasectomy. (**b**) Overall survival after third pulmonary metastasectomy.

Seventeen patients underwent third PM, 5 patients underwent fourth PM, 2 patients underwent fifth PM and 1 patient underwent sixth PM. The characteristics of the third PM are presented in Table 5. One patient (6%) experienced postoperative complications (atrial fibrillation). After the third PM, 13 (76%) patients experienced recurrence. At the time of writing this report, 6 patients were alive without disease, 1 patient was alive with disease and 10 patients died. The 3-year RFS and OS rates after the third PM for patients who underwent the third PM (*n* = 17) were 22% and 53%, respectively (Fig. 3a/b).

**Table 5:** Characteristics at third pulmonary metastasectomy

Characteristics	Third PM (*n* = 17)^a^
Age (years)	
Mean ± SD	38.2 ± 19.0
Range	16–79
Primary tumour, *n* (%)	
Soft tissue sarcoma	7 (41%)
Osteosarcoma	6 (35%)
Colorectal cancer	3 (18%)
Head and neck cancer	1 (6%)
Laterality, *n* (%)	
Unilateral	15 (88%)
Bilateral	2 (12%)
Number of lesions, *n* (%)	
1	15 (88%)
2	2 (12%)
Size of pulmonary metastasis (cm)	
Mean ± SD	2.4 ± 1.7
Range	0.4–7.2
Type of resection, *n* (%)	
Lobectomy or bilobectomy	3 (19%)
Segmentectomy	3 (19%)
Partial resection	10 (62%)
Operation time (min), mean ± SD	136 ± 65
Blood loss (g), mean ± SD	207 ± 553
Complete resection, *n* (%)	15 (88%)
Postoperative complications, *n* (%)	1 (6%)

a In 1 patient who underwent two-stage pulmonary resection for bilateral metastases, data from the preceding pulmonary resection procedure were used.

PM: pulmonary metastasectomy; SD: standard deviation.

The details of the 6 patients who eventually had no evidence of disease are shown in Supplementary Material, Table S2. Of note, 3 out of 17 patients (18%) who underwent the third PM achieved 3-year disease-free survival.

## DISCUSSION

In this study, the 5-year RFS and OS rates after the second PM were 27% and 48%, respectively. The interval between the first PM and subsequent detection of pulmonary metastasis (≤12 months) was identified as a poor prognostic factor for both RFS and OS after the second PM. Seventeen patients underwent a third PM, of whom 3 (18%) achieved 3-year disease-free survival.

Repeat PM has been conducted in various types of cancers, with a particularly large number of reports concerning sarcoma and CRC. Reported rates of patients who undergo a second PM among those who have already undergone the first PM are 26–43% in sarcoma and 15–19% in CRC [15–18]. Many reports have demonstrated favourable outcomes after the second PM. In terms of postoperative complications, Forster *et al.* [19] reported that the postoperative cardiopulmonary complication rate and median length of stay were not significantly different between first and second PM. In terms of long-term outcomes, the survival of patients who underwent a second PM is reportedly equivalent to or better than that of patients who underwent the first PM alone [20]. Furthermore, the survival after the second PM (calculated from the second PM) is reportedly almost the same as or better than that after the first PM (calculated from the first PM) [17, 21]. Some reports showed repeat resection to be a good prognostic factor in patients who underwent first PM [18, 22]. In the present study, the 5-year RFS and OS rates after second PM were 27% and 48%, respectively. Sixteen patients did not experience any recurrence after the second PM. Of these, 13 patients received no additional treatment, and 8 achieved 5-year disease-free survival. Based on these observations, it is considered that an almost ‘cured’ status can be achieved at a certain rate with repeated PM.

There is no firm evidence of the effectiveness of repeat PM, other than observational studies. Fiorentino and Treasure [23] argued on this issue, maintaining that reselection of the most favourable patients for repeat PM is the likely reason for any differences in survival between the initial and subsequent PM procedures. In addition to PM, stereotactic body radiation therapy and radiofrequency ablation are currently available local treatment options for pulmonary metastasis. However, while these treatments provide a favourable local control rate, the comparison of PM and these treatments is difficult because the indications for these treatments are generally limited to patients who cannot tolerate surgery [24, 25]. The present study demonstrated that the survival after pulmonary recurrence after the first PM was significantly better in patients with a second PM group than in those without a second PM (5-year OS: 53% vs 41%, *P* = 0.01). However, it should be noted that this difference does not directly mean that repeat PM is a better mode of treatment than other treatments.

In this situation, retrospective studies comparing repeat PM and nonsurgical treatment using well-matched controls are currently the best evidence available. Chudgar *et al.* conducted a weight-based propensity score-matched analysis of 341 STS patients who experienced pulmonary recurrence after the first PM. Even after controlling for the characteristics of the primary tumour and metastatic disease, the survival of patients who underwent repeat PM was still significantly better than that of those who underwent nonsurgical treatment [15]. Hishida *et al.* [18] analysed data from 216 patients who experienced limited lung recurrence after initial resection of pulmonary metastases from CRC. In their study, 132 (61%) patients underwent repeat lung resection, and their 5-year OS rate was 75.3% while that of the patients who did not undergo repeat lung resection was 23.3%. Furthermore, as a result of multivariable analyses of factors predicting survival in patients with lung-limited recurrence after first PM for metastasis from CRC, repeat lung resection was identified as an independent predictor of better survival in their study. Based on the findings of previous studies and our own experience, repeat PM is currently regarded as a first-choice treatment for patients who develop pulmonary recurrence after PM and who meet the widely used criteria for PM [4].

In the present study, it was demonstrated that the interval between the first PM procedure and subsequent detection of pulmonary metastasis (≤12 months) was a poor prognostic factor for both RFS and OS after the second PM. Although substantial information has been gathered regarding prognostic factors after the first PM, data on prognostic factors after the second PM are limited [2]. Kandioler *et al.* [26] conducted a study on prognostic factors after the second PM, reporting that a disease-free interval (DFI) >1 year was significantly associated with a survival advantage beyond the last operation. Reports on prognostic factors after the second PM published after 2016 are shown in Supplementary Material, Table S3 [15, 18, 20, 27, 28]. Four reports included CRC patients, and 1 included STS patients. Among them, Ihn *et al.* [27] analysed the outcomes of 39 patients with CRC who underwent a second PM and showed that a recurrent DFI of <12 months was a poor prognostic factor for OS. Given these present and previous findings, patients with DFI >12 months are expected to demonstrate favourable long-term outcomes and are thus considered good candidates for a second PM.

To date, there have been few reports of third PM. In the present study, the site of relapse was pulmonary metastasis alone in 30 out of 52 patients (58%) who experienced relapse after the second PM, suggesting that some patients suffer from pulmonary metastasis alone and no other site of relapse, so even a third PM can be performed in select patients. Reportedly, only 5% of patients who undergo PM initially undergo the procedure ≥3 times [11]. Recently, Mills *et al.* [13] analysed the short-term outcomes of 117 patients who underwent a third PM (60 patients with sarcoma and 37 with CRC). In their report, the estimated blood loss did not differ markedly between the first and second PM procedures; however, it significantly increased during the third procedure. The rate of wound complications at the third PM was also significantly higher than that at the second PM, and the likelihood of prolonged air leakage increased incrementally in each subsequent operation. In the present study, the operation time tended to be longer, blood loss tended to be higher and the rate of postoperative complications tended to be higher in the second PM group than in the first PM group. In terms of the third PM, operation time and blood loss were not markedly different from those at the second PM, and the rate of postoperative complications was lower than that at the second PM. This observation in our study may be attributed to differences in patient characteristics, that is, patients who underwent a third PM were younger than those who underwent a second PM and thus had fewer comorbidities.

To our knowledge, there has been little information on the mid- to long-term outcomes after third PM. Krüger *et al.* [12] reported the outcomes of repeat PM from a multicentre trial. In their report, a total of 621 patients underwent first PM, and of these, 64 patients underwent a second PM, 35 underwent a third PM, 12 underwent a fourth PM and 6 underwent a fifth PM. They reported favourable long-term outcomes of repeat PM, with the following 5-year survival rates after each procedure: first PM, 63.3%; second PM, 50.9%; third PM, 74.4%; fourth PM, 83.3%; and fifth PM, 60.0%. In this study, 17 patients underwent a third PM procedure. As shown in Supplementary Material, Table S2, 6 of these patients eventually had no evidence of disease, and 3 patients (18%) achieved a 3-year disease-free survival. Although the number of patients was very small, the third PM seems beneficial in certain select patients, based on our experience. Based on Krüger’s report and our own experience, it is considered that third PM can be regarded as a viable treatment option for recurrent pulmonary metastasis.

### Limitations

Several limitations of the present study warrant mention. First, the study analysed patients who were treated for over 2 decades, during which there were some advances in radiological and therapeutic modalities. In particular, outcomes of PM were largely affected by changes in modalities for assessing extrapulmonary metastases. Second, the follow-up period was relatively short (median, 36 months). Third, this was a retrospective single-center analysis and the number of patients was limited. This study is associated with the inherent limitations of retrospective studies, and it would be ideal to conduct a multivariable analysis in a large cohort. To clarify the prognostic factors more precisely, further multicentre studies with larger patient numbers are needed.

## CONCLUSION

Patients with a period of >12 months between the first PM and the subsequent detection of pulmonary metastases showed favourable outcomes and are thus considered good candidates for second PM. A third PM may be beneficial for selected patients.

## Supplementary Material

ivae028_Supplementary_Data

## Data Availability

The datasets used and/or analysed during the current study are available from the corresponding author upon reasonable request.

## ABBREVIATIONS

CRC: Colorectal cancer
CT: Computed tomography
OS: Overall survival
PM: Pulmonary metastasectomy
RFS: Relapse-free survival
STS: Soft tissue sarcoma
