# Supplementary material for: Outcomes and prognostic factors of repeat pulmonary metastasectomy
Source: Interdiscip Cardiovasc Thorac Surg. 2024 Feb 29;38(3):ivae028. doi: 10.1093/icvts/ivae028 (PMC10927334; doi:10.1093/icvts/ivae028)
Supplement: ivae028_Supplementary_Data [file ivae028_supplementary_data.zip › supplementary Fig 1.pdf]

Supplementary Figure 1

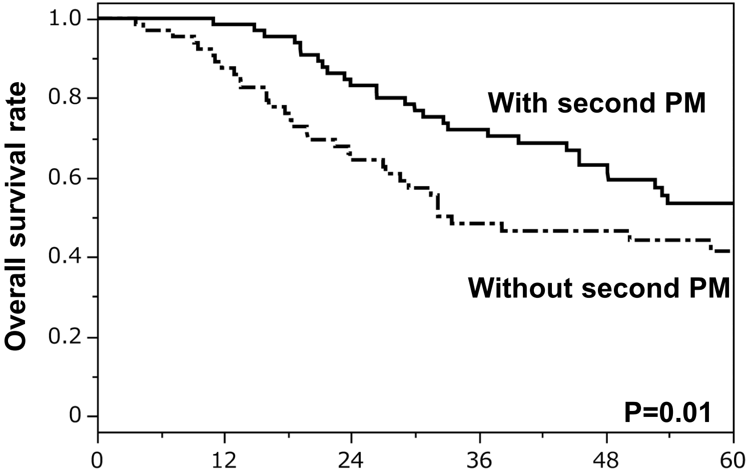

Number of Patients at risk						
With second PM	68	65	54	44	34	26
Without second PM	68	55	38	26	21	15
