# Supplementary material for: Outcomes and prognostic factors of repeat pulmonary metastasectomy
Source: Interdiscip Cardiovasc Thorac Surg. 2024 Feb 29;38(3):ivae028. doi: 10.1093/icvts/ivae028 (PMC10927334; doi:10.1093/icvts/ivae028)
Supplement: ivae028_Supplementary_Data [file ivae028_supplementary_data.zip › 231014supplementaryfigurelegend.docx]

**Supplementary Figure 1.** The overall survival of 68 patients underwent second pulmonary metastasectomy (with second PM group) and 68 patients experienced lung-limited recurrence and did not undergo second pulmonary metastasectomy (without second PM group) after detection of pulmonary recurrence after the first pulmonary metastasectomy. The survival after pulmonary recurrence after the first pulmonary metastasectomy was significantly better in with second PM group than that of without second PM group. (5-year OS: 53% vs. 41%, p=0.01). PM, pulmonary metastasectomy.
