# Supplementary material for: Outcomes and prognostic factors of repeat pulmonary metastasectomy
Source: Interdiscip Cardiovasc Thorac Surg. 2024 Feb 29;38(3):ivae028. doi: 10.1093/icvts/ivae028 (PMC10927334; doi:10.1093/icvts/ivae028)
Supplement: ivae028_Supplementary_Data [file ivae028_supplementary_data.zip › 231014supplementarytablesunmarked.docx]

**Supplementary Table 1. The primary tumor of 549 patients who underwent first pulmonary metastasectomy.**

| Type of primary tumor | n=549 |
| --- | --- |
| Colorectal cancer | 192 (35%) |
| Head and neck cancer | 74 (13%) |
| Soft tissue sarcoma | 74 (13%) |
| Breast cancer | 37 (7%) |
| Renal cell carcinoma | 31 (6%) |
| Uterine cancer | 25 (5%) |
| Osteosarcoma | 22 (4%) |
| Pancreatic cancer | 17 (3%) |
| Transitional cell carcinoma of urinary tract | 15 (3%) |
| Oesophageal cancer | 14 (3%) |
| Germ cell tumor | 11 (2%) |
| Hepatocellular carcinoma | 10 (2%) |
| Thyroid cancer | 6 (1%) |
| Gastric cancer | 5 (1%) |
| Others | 16 (3%) |

**Supplementary Table 2. Details of six patients who eventually had no evidence of disease.**

| Case | Primary disease | Age (years) at treatment for primary tumor | Distant metastasis besides lung throughout the clinical course | Treatment after third PM | Total number of pulmonary metastases resected throughout the clinical course | Time after treatment for primary tumor (mo) | Time after the last local treatment for pulmonary metastasis (mo) |
| --- | --- | --- | --- | --- | --- | --- | --- |
| 1 | STS | 14 | no | none | 4 | 277 | 195 |
| 2 | Ost | 18 | no | 4th and 5th PM for pulmonary metastasis | 10 | 177 | 99 |
| 3 | CRC | 66 | no | none | 3 | 95 | 36 |
| 4 | STS | 77 | no | none | 4 | 49 | 28 |
| 5 | CRC | 43 | yes | none | 5 | 66 | 20 |
| 6 | CRC | 63 | yes | RFA for pulmonary metastasis | 4 | 111 | 11 |

CRC, colorectal cancer; Ost, osteosarcoma; PM, pulmonary metastasectomy; RFA, radiofrequency ablation; STS, soft tissue sarcoma.

**Supplementary Table 3. Reports on prognostic factors after second PM published after 2016.**

| Author | Year | Study Period | Number of patients | Type of primary tumor | Poor prognostic factors for the OS after second PM |
| --- | --- | --- | --- | --- | --- |
| Hachimaru | 2016 | 2004-2013 | 33 | CRC | elevated preoperative serum CEA level |
| Hishida | 2017 | 2004-2008 | 132 | CRC | concomitant liver metastasis, location of primary tumor in rectum |
| Ihn | 2017 | 2003-2011 | 39 | CRC | recurrent DFI < 12 months |
| Chudgar | 2017 | 1991-2014 | 141 | STS | leiomyosarcoma, preoperative chemotherapy, incomplete resection |
| van Dorp | 2023 | 2012-2019 | 127 | CRC | DLco < 80% |
| Our study | 2023 | 2000-2020 | 68 | Various | Interval between first PM and subsequent detection of pulmonary metastasis ≤ 12 |

CEA, Carcinoembryonic Antigen; CRC, colorectal cancer; DFI, disease-free interval; DLco, Diffusing capacity of the lungs for carbon monoxide; OS, overall survival; PM, pulmonary metastasectomy; STS, soft tissue sarcoma.
